# New frontiers of retinal therapeutic intervention: a critical analysis of novel approaches

**DOI:** 10.1080/07853890.2022.2066169

**Published:** 2022-04-25

**Authors:** Onnisa Nanegrungsunk, Adrian Au, David Sarraf, Srinivas R. Sadda

**Affiliations:** a Doheny Eye Institute, Pasadena, CA, USA; b Department of Ophthalmology, David Geffen School of Medicine, University of California-Los Angeles, Los Angeles, CA, USA; c Retina Division, Department of Ophthalmology, Faculty of Medicine, Chiang Mai University, Chiang Mai, Thailand; d Stein Eye Institute, David Geffen School of Medicine, University of California-Los Angeles, Los Angeles, CA, USA

**Keywords:** Advanced therapy, anti-vascular endothelial growth factor (anti-VEGF), artificial vision, complement cascade targeted therapy, dual-target therapy, gene therapy, optogenetics gene therapy, port delivery system, retinal prosthesis, stem cell therapy

## Abstract

A recent wave of pharmacologic and technologic innovations has revolutionized our management of retinal diseases. Many of these advancements have demonstrated efficacy and can increase the quality of life while potentially reducing complications and decreasing the burden of care for patients. Some advances, such as longer-acting anti-vascular endothelial growth factor agents, port delivery systems, gene therapy, and retinal prosthetics have been approved by the US Food and Drug Administration, and are available for clinical use. Countless other therapeutics are in various stages of development, promising a bright future for further improvements in the management of the retinal disease. Herein, we have highlighted several important novel therapies and therapeutic approaches and examine the opportunities and limitations offered by these innovations at the new frontier.

**KEY MESSAGES**
- Numerous pharmacologic and technologic advancements have been emerging, providing a higher treatment efficacy while decreasing the burden and associated side effects.
- Anti-vascular endothelial growth factor (anti-VEGF) and its longer-acting agents have dramatically improved visual outcomes and have become a mainstay treatment in various retinal diseases.
- Gene therapy and retinal prosthesis implantation in the treatment of congenital retinal dystrophy can accomplish the partial restoration of vision and improved daily function in patients with blindness, an unprecedented success in the field of retina.

## Introduction

Robust commitment and investment in novel research have allowed the development of novel therapeutics which offer the potential to dramatically improve visual outcomes for patients and reduce blindness worldwide. Many of these recent innovations offer better treatment efficacy and higher quality of life for patients. They restore lost vision while minimizing the side effects and burden related to the treatment. Herein, we introduce and highlight some of the newest retinal therapeutics and discuss their advantages and limitations as they usher in a new frontier of novel retinal therapeutics.

## Methods

To compile this review article, English language articles were retrieved using a keyword search in PubMed in August 2021 and then supplemented by manual searching of references of articles published online through November 2021. In addition, recent clinical trials for a retinal disease listed on clinicaltrials.gov from 2015 to 2021 as well as press releases of clinical trials results were reviewed in cases where published articles for a therapeutic were not available.

## Results

Over 100 English articles regarding novel therapeutics in retinal diseases were reviewed and summarized into six major topics: single-target therapy, dual-target therapeutics, technology-based approaches for increasing drug durability and delivery, gene therapy, stem cell therapy, and artificial vision.

### Single-target therapy

#### Anti-vascular endothelial growth factor and its longer durability agents

Since 2004 when anti-vascular endothelial growth factor (anti-VEGF) was introduced to treat eye diseases, anti-VEGF has become the mainstay of treatment for many retinal diseases including neovascular (wet) age-related macular degeneration (nAMD), diabetic macular edoema (DME) and macular edoema due to retinal vein occlusion (RVO) [1–7]. Intravitreal anti-VEGF therapy inhibits the growth of neovascularization, reduces fluid leakage, and is superior to previously-available treatments, such as corticosteroids, laser photocoagulation, or photodynamic therapy [8–12]. Anti-VEGF therapy not only prevents vision loss but also restores or improves vision in many cases. Pegaptanib, an RNA aptamer, was the first anti-VEGF agent approved for ophthalmic use by the US Food and Drug Administration (FDA) in 2004, but only blocks the 165-isoform of VEGF. While it was shown to reduce moderate vision loss associated with nAMD, on average, patients did not improve vision, and thus its use was limited compared to other later-approved anti-VEGF therapies [13]. Other agents that are currently available within the United States include ranibizumab (Lucentis; Novartis, East Hanover, NJ, USA/Genentech, South San Francisco, CA, USA), bevacizumab (Avastin; Genentech; off-label use), aflibercept (Eylea; Regeneron Pharmaceuticals, Tarrytown, NY, USA) and most recently brolucizumab (Beovu; Novartis).

Ranibizumab is an antibody fragment that binds to all isoforms of VEGF-A and received FDA approval in 2006. Bevacizumab is a full antibody against VEGF-A and has been extensively used off-label because of its cheaper availability. Aflibercept is a recombinant fragment crystallizable (Fc) fusion protein against VEGF-A, VEGF-B, and placental growth factor (PIGF) and received FDA approval in 2011. Brolucizumab is a single-chain variable fragment antibody against VEGF-A and received FDA approval in 2019. Although ranibizumab and bevacizumab have demonstrated good efficacy with visual gains of at least 5–10 letters in the treatment of various retinal diseases, monthly injections or frequent monitoring may be required to achieve that effectiveness [2,4,5,7]. Aflibercept and especially brolucizumab offer longer drug durability, and an injection interval of every 2–3 months after serial loading doses is possible in many eyes [3,14,15]. During year 2 of the intravitreal aflibercept study in nAMD (VIEW 1 and 2; NCT00509795 and NCT00637377), participants were allowed to defer the injection interval (not longer than 12 weeks) using an as-needed (*pro re nata*; PRN), or “capped-PRN” regimen. The study showed that ∼92% of participants maintained vision at year 2 with an average of 7.6 letters gain with this aflibercept capped-PRN regimen. Approximately, one letter was lost compared to year 1 when transitioning from fixed-dosing to the capped-PRN regimen, while the number of injections was reduced from 7–12 injections in year 1 to 4 injections during the capped-PRN in year 2 [3]. The intravitreal brolucizumab arm demonstrated six letters gained with an 8- or up to 12-week dosing regimen in nAMD (HAWK and HARRIER; NCT02307682 and NCT02434328) after 2 years, with the superior resolution of exudation compared with the aflibercept arm [15]. However, cases of intraocular inflammation and occlusive retinal vasculitis following intravitreal brolucizumab injection have been reported which led to significant visual loss in a few eyes. A careful examination for inflammation after brolucizumab injection and discussion with the patient regarding these risks is recommended [16].

Other anti-VEGFs that have been used include ziv-aflibercept (Regeneron/Sanofi, Bridgewater Township, NJ, USA) and conbercept (Chengdu KangHong Biotech, Sichuan, China). However, as of yet, these two agents have not been approved by the FDA in the United States but are available in other countries [14]. Abicipar pegol (Allergan; Dublin, Ireland) is another longer durability agent comprised of ankyrin repeat proteins (DARPins) against VEGF-A. The Phase III study of abicipar pegol showed visual improvement and non-inferiority to monthly ranibizumab, with fewer injections as the frequency of intravitreal abicipar injection could be extended up to every 12 weeks [17]. The drug however has not received FDA approval, due to the significant risk of severe intraocular inflammation [18].

Biosimilars, unlike generics, are biological products that require a more complex manufacturing process and therefore require a more robust FDA-approval process to ensure safety and equivalency *vs.* the “reference product”. Biosimilars provide safe, lower-cost options for the treatment of retinal diseases while providing equal efficacy. Ranibizumab biosimilars include SB11 (Samsung Bioepis, Incheon, Republic of Korea) and FYB201 (Bioeq GmbH, Holzkirchen, Bayern, Germany), and Phase III trials have shown efficacy and safety equivalent to the reference biologic in the treatment of nAMD [19,20]. Aflibercept biosimilars include ABP938 (Amgen, Thousand Oaks, CA, USA; NCT04270747), FYB203 (Bioeq GmbH; NCT04522167), and SB15 (Samsung Bioepis; NCT04450329) and are also being investigated in Phase III trials for nAMD. Recently, the ranibizumab biosimilar SB11 (Byooviz; Samsung Bioepis) was approved by the FDA (2021) for the treatment of nAMD, macular edoema following RVO, and myopic choroidal neovascularization [21].

#### Complement cascade targeted therapy

The role of the immune system and inflammation has been studied in age-related macular degeneration (AMD) and diabetic retinopathy (DR) and evidence suggest that the complement system is active in diseases with chronic inflammation and contributes to the pathology of these disorders [22]. There are three main pathways of activation of the complement cascade: the classical pathway, mannose-binding lectin pathway, and alternative pathway. All three pathways converge at the point of cleavage of C3 and then C5, leading to the formation of other fragments e.g. C3a, C3b, C5a, C5b-9, which then induce inflammation, vasodilation, permeability of small vessels, and immune regulation. Evidence of activity of the complement system has been observed at the level of the retina, retinal pigment epithelium (RPE), and choroid [22]. Various complement components have been identified in drusen in AMD and in the vitreous of proliferative diabetic retinopathy (PDR) eyes [23,24].

Agents targeting complement have been investigated over the last decade. Preliminary results of these agents have generally demonstrated a good safety profile in humans. However, the true efficacy in the treatment of retinal diseases is still under investigation and varies among agents. For example, the pivotal clinical trials of lampalizumab (Hoffmann-la Roche, Basel, Switzerland), an anti-factor D antibody, failed to meet the primary end point as the lampalizumab arm did not slow the progression of geographic atrophy (GA) from baseline *vs.* the control arm (NCT02247479 and NCT02247531). A Phase II/III study of avacincaptad pegol (Zimura; IVERIC bio Inc., New York, NY, USA), a C5 inhibitor, showed a 27.4% reduction in the mean rate of GA growth over 12 months (*p* = .007) in the 2-mg avacincaptad pegol arm and a reduction of 27.8% (*p* = .005) in the 4-mg avacincaptad pegol arm, compared to sham [25]. Pegcetacoplan (APL2-103; Apellis Pharmaceuticals Inc., Waltham, MA, USA), a C3 inhibitor, also showed a reduction in GA growth rate by 22% (*p* = .0003) in monthly treated subjects and 16% (*p* = .005) in the every-other-month treated group, compared with sham at month 12 in one of the Phase III trials (OAKS; NCT03525600). However, the parallel Phase III trial (DERBY; NCT03525613) did not demonstrate a statistically significant reduction in GA growth with reduced rates of 12% (*p* = .05) and 11% (*p* = .07) in the monthly and every-other-month groups, respectively, compared to control. In a prespecified analysis of combined OAKS and DERBY, monthly and every-other-month treatment with pegcetacoplan groups reduced GA lesion growth by 17% (*p*<.0001) and 14% (*p* = .0012), respectively, compared to pooled sham at 12 months. Thirteen cases of intraocular inflammation (0.21% per injection) and three cases of infectious endophthalmitis (0.05% per injection) were reported. The pooled rate of new-onset exudation, including those detected by the reading centre, was 6.4% of patients in the monthly pegcetacoplan group, 5.0% in the every-other-month pegcetacoplan group, and 3.8% in the sham group [26,27]. Some complement inhibitors, while unsuccessful for retinal disease, have demonstrated systemic efficacy. For example, systemic eculizumab (Alexion Pharmaceuticals [Cheshire, CT]), an anti-C5 antibody, did not significantly decrease the growth rate of GA secondary to AMD, but significantly lowered the risk of relapse in aquaporin-4 antibody (AQP4-IgG) positive neuromyelitis optica spectrum disorder (NMOSD) [28,29].

With the promise of previous results, clinical trials investigating avacincaptad pegol are fully enrolled in the second Phase III study in AMD with anticipated results due to be released in the near future, and Phase II trials are underway to study hereditary retinal diseases (NCT03364153). LFG316 (Novartis) is a C5 antibody and is being investigated in a recently completed Phase II study (NCT01527500). The efficacy of complement cascade therapy combined with other agents, e.g. anti-VEGF drugs, is also being conducted. The study of avacincaptad pegol combined with ranibizumab in nAMD is completed and the results are anticipated to be released soon (NCT03362190).

### Dual-target therapeutics

Faricimab (RG7716; Genentech) is a first-in-class bi-specific monoclonal antibody that targets both VEGF-A and angiopoiten-2 (Ang-2). Ang-2 is a context-dependent agonist and antagonist of TIE2 receptor tyrosine kinase that is associated with vascular remodelling. During inflammation, Ang-2 acts as an antagonist. It promotes endothelial cell permeability, increases VEGF-A dependent NV, and stimulates pericyte apoptosis [30,31]. Ang-2 levels have been found to be elevated in common retinal vascular diseases including nAMD, DR, and RVO [32–34]. The Fc region of faricimab was also designed for faster systemic clearance, reducing the risk for potential inflammatory adverse events [35]. Phase I/II clinical trials of faricimab investigated the safety and efficacy of this agent in these retinal diseases [36–38]. In Phase III clinical trials of faricimab in nAMD (TENAYA and LUCERNE; NCT03823287 and NCT03823300), the average vision gains compared with baseline in the faricimab arms (fixed-dosing intervals of every 2, 3, or 4 months) were 5.8 and 6.6 letters, respectively, compared to 5.1 and 6.6 letters in the fixed-dosing aflibercept every 2 months (8 weeks) arms. In addition, 46% of patients in TENAYA and 45% in LUCERNE receiving faricimab were treated every 16 weeks in the first year [39].

For DME, the YOSEMITE (NCT03622580) and RHINE (NCT03622593) trials showed average vision gains of 10.7 and 11.8 letters in the every 8-week faricimab group, and 11.6 and 10.8 letters in the faricimab personalized treatment interval (PTI) group, compared with 10.9 and 10.3 letters in the every 8-week aflibercept group, respectively. Moreover, 53% of patients in YOSEMITE and 51% of patients in RHINE achieved every 16 week dosing per 1-year [39]. Clinical trials are in progress, evaluating the efficacy and durability of faricimab in the treatment of RVO (COMINO and BALATON; NCT04740931 and NCT04740905) compared with aflibercept.

Overall, faricimab appears to be non-inferior to existing anti-VEGF-based treatments, such as aflibercept, but is also associated with a longer injection-free interval as approximately half of the patients that received faricimab were able to defer injection to every 16 weeks.

Pegpleranib (Fovista; Ophthotech, New York, NY, USA), a DNA aptamer that binds to platelet-derived growth factor (PDGF) receptors on pericytes, was believed to offer the potential to strip neovascular pericytes from the underlying endothelial cells, increasing their vulnerability and sensitivity to VEGF blockage [40]. However, the Phase III trial of pegpleranib in combination with anti-VEGF therapy (aflibercept or bevacizumab) compared to anti-VEGF monotherapy (NCT01940887) showed no statistically significant visual gain at 12 months (9.42 letters in the combination therapy group *vs.* 9.04 letter in the anti-VEGF monotherapy group; difference, 0.38 letters; *p* = .74) [41].

### Technology-based approaches for increasing drug durability and delivery

#### Port delivery system

**Figure 1.**
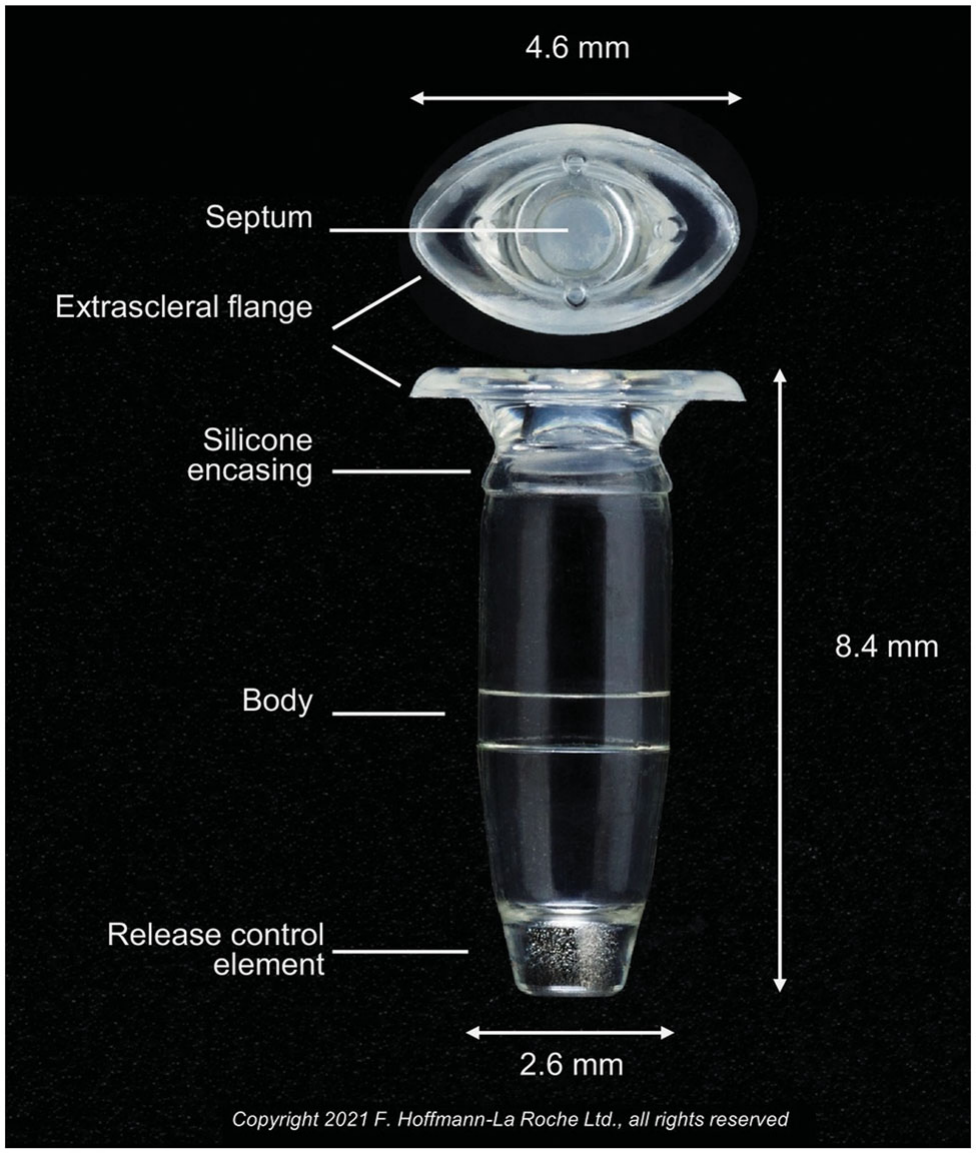
Figure of the port delivery system (PDS) implant with dimensions of various components as shown. Copyright 2021 F. Hoffmann-La Roche Ltd., all rights reserved. Used with permission.

**Figure 2.**
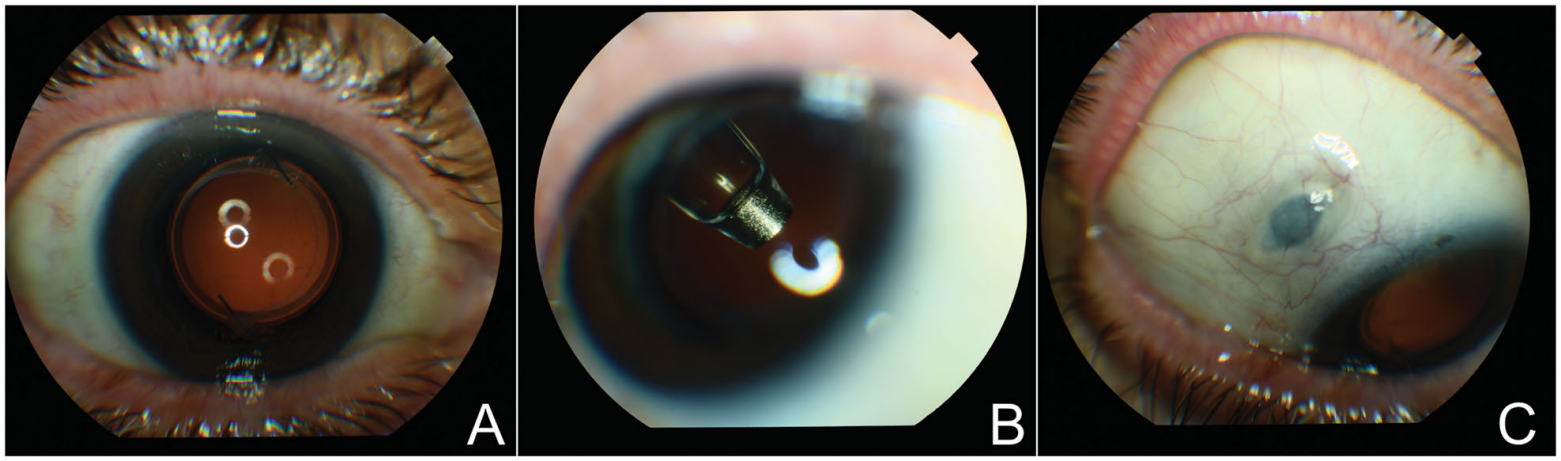
Clinical images from PDS-implanted patients. (A) In the primary position, the PDS implant is not visible through the dilated pupil. (B) In superotemporal gaze, the lower portion of the body of the implant and the release control element are visible through the dilated pupil. (C) When eye looking inferonasally, the septum of the implant is visible through the conjunctiva. Images courtesy of Dr. Arshad Khanani, Sierra Eye Associates, Reno, NV, USA.

The port delivery system with ranibizumab (PDS; Genentech/Roche) is a small refillable eye implant that continuously delivers ranibizumab into the eye (Figures 1, 2). This is in contrast to a single intravitreal injection of ranibizumab 0.5 mg which is delivered into the eye in a bolus fashion. With most retinal diseases, intravitreal injections may need to be repeated monthly, especially in the first year of treatment [2,5,42]. With the 2 mg-reservoir of PDS, ranibizumab is slowly released into the eye and the ranibizumab level remains within the therapeutic range after 6 months of implantation. The Phase III trial of PDS with ranibizumab in nAMD patients (Archway Study; NCT03677934) showed that 242 of 246 patients (98.4%) in the PDS cohort did not receive any supplemental treatment or injection before the first refill or exchange (fixed at every 6 months), indicating PDS with ranibizumab could reach the target duration. Small visual gains were noted in each arm and were not significantly different (0.2 letters in the PDS-treated arm *vs.* 0.5 letters in the monthly ranibizumab arm; adjusted mean difference, −0.3 letters; 95% CI, −1.7–1.1) as the study eyes were treated with at least 3 anti-VEGF intravitreal injections and demonstrated evidence of anatomical and visual response to anti-VEGF treatment before enrolment. The PDS group also maintained visual acuity at a level comparable to the monthly ranibizumab injection group, and ∼80% of both groups demonstrated visual acuity of 20/40 or better over 36 to 40 weeks after treatment. Forty-seven (19.0%) PDS-treated patients developed ocular adverse events including 4 patients (1.6%) with endophthalmitis, 2 patients (0.8%) with rhegmatogenous retinal detachment, and 13 patients (5.2%) with vitreous haemorrhage which spontaneously resolved [43–45]. Of the total of 450 implants that were performed in the PDS trials, 6 implant dislocations occurred but these were all encountered before the updated implant procedure and with a scleral incision >3.7 mm [44]. Recently, PDS has received FDA approval for the treatment of nAMD (October 2021). Studies of PDS in DME (PAGODA; NCT04108156) and DR (PAVILION; NCT04503551) are currently ongoing.

#### Genetically engineered cell line with encapsulated cell technology

NT-501 (Neurotech Pharmaceuticals, Cumberland, RI, USA) is a genetically modified human RPE cell line that produces ciliary neurotrophic factor (CNTF). CNTF can slow vision loss and retard photoreceptor death and degeneration of cone outer segments in animal models [46,47]. The modified RPE cells are encapsulated in a semipermeable membrane designed for long-term drug delivery that allows for diffusion of CNTF into the eye while masking the cells from the host immune system. A Phase II trial of the NT-501 implant was conducted in macular telangiectasia type 2 patients. The primary end point was a change in the area of photoreceptor ellipsoid zone (EZ) loss as assessed with cross-sectional spectral-domain optical coherence tomography. Eyes in the sham group displayed a larger area of EZ loss at 24 months (difference, 0.05 ± 0.03 mm^2^; *p* = .04) with 31% greater progression of neurodegeneration than in the implant group [48]. A Phase III study of NT-501 in macular telangiectasia type 2 patients (NCT03316300) is currently ongoing and the studies are expanding to other diseases, such as glaucoma (NCT04577300).

### Gene therapy

#### Adeno-associated virus-based gene therapy

One of the major achievements in the modern era of medicine is the sequencing of the human genome [49]. Since its completion, the field of genetics has revolutionized our collective understanding of genes and their impact on human disease and has made major breakthroughs in the development of gene-based treatments. This is best illustrated with the first adeno-associated virus (AAV)-based gene therapy in the treatment of mutated retinoid isomerohydrolase 65 (RPE65) in Leber congenital amaurosis-2 (LCA-2) [50]. RPE65 is the critical enzyme expressed in photoreceptors that helps convert all-trans-retinal to 11-cis-retinal, an important step in the visual cycle [51]. LCA patients usually present with reduced vision and nyctalopia as a consequence of retinal degeneration between birth and five years of age [52]. Preclinical animal studies in mice and canines harbouring the RPE65 mutant showed visual rescue by subretinal injections of AAV-RPE65 [53,54].

In the pivotal Phase III randomized controlled clinical trial, bilateral subretinal voretigene neparvovec-rzyl (Luxturna; AAV2-hRPE65v2; Spark Therapeutics [Philadelphia, PA]/Roche) administration was evaluated for safety and efficacy in patients with RPE65-mediated inherited retinal dystrophy [50]. To determine whether patients improved, a novel standardized multi-luminance mobility test (MLMT) was created to overcome the limitations of traditional cone-based central visual acuity testing for evaluating the treatment of a rod-based disease. The results of the study demonstrated an improvement of 1.8 ± 1.1 MLMT scores in the intention-to-treat group *vs.* 0.2 ± 1.0 in the control group (difference, 1.6; 95% CI, 0.72–2.41; *p* = .0013) at one year follow-up [50]. Thirteen of twenty patients (65%) passed the lower luminance level tested (1 lux) while no control participants passed the MLMT at 1 lux at 1 year. There was a non-statistically significant visual improvement from baseline between treated (−0.16 LogMAR) *vs.* control (−0.01 LogMAR) groups (adjusted for modelled mean changes of both groups; difference, −0.16 LogMAR; 95% CI, −041–0.08; *p* = .17). Durability was later confirmed at 4 years at which point treated patients retained an MLMT score of 1.7 and 71% of patients were able to pass at the lowest light level [55]. No serious adverse events related to the therapy or deleterious immune responses were noted at 1 year but one patient developed a retinal detachment at year 4. These findings allowed voretigene neparvovec-rzyl to become the first genetic therapy approved by the FDA for the treatment of patients with confirmed RPE65 mutation-associated retinal dystrophy in 2017.

Another viral-based gene therapy under consideration targets choroideremia, an X-linked recessive choroidal dystrophy caused by a mutation in the CHM gene. The CHM gene encodes a Rab escort protein 1 (REP1) which helps facilitate intracellular protein trafficking *via* prenylation and membrane expression of the Rab protein [56]. Although ubiquitously expressed, patients with choroideremia can develop rapid atrophy of the choroid, RPE, and outer retina causing nyctalopia generally by the third decade but with retained central vision as the macula may be spared [57]. Pre-clinical trials in animal and cell cultures demonstrated a rescued phenotype when ectopically expressing CHM [58–61]. In humans, the first clinical trial conducted by the University of Oxford (NCT01461213) enrolled 14 patients and showed a median visual gain of 5.5 ± 6.8 letters after subretinal injection of AAV.REP1 vector at 2 years [62,63]. However, one patient developed iatrogenic stretching of the retina, and another developed intraocular inflammation. Subsequent clinical trials from Tubingen (NCT02671539), Alberta (NCT02077361), Miami (NCT02553135), and Philadelphia (NCT02341807) enrolled 40 patients. The aggregated 2-year visual outcome in 34 patients revealed a median gain of 1.5 ± 7.2 Snellen letters (−14 min, 18 max) across all trials [64–67]. The major complications were related to the subretinal injection procedure including one patient with a retained residual air bubble in the subretinal space and another with foveal thinning after the injection. Other common side effects, such as mild post-op inflammation resolved with systemic or topical steroids. Despite the marginal improvement of vision and the variability of outcomes between trials, the promising results of the early clinical trials in the management of choroideremia highlight the prospects for genetic therapy in diseases beyond RPE65 mutation-mediated LCA. Results of 4 other ongoing Phase II or Phase III clinical trials (NCT02407678, NCT03507686, NCT03496012, and NCT04483440) are pending.

Gene therapy in nAMD has also been studied as an approach to reduce the burden of anti-VEGF injections. Two leading investigational therapeutic strategies are focussed on achieving an anti-VEGF effect by expressing currently used monoclonal antibodies: ranibizumab (RGX-314; REGENXBIO Inc., Rockville, MD, USA) or aflibercept (ADVM-022; Adverum Biotechnologies, Redwood City, CA, USA). In Phase I/II trials of subretinal injection RGX-314 that utilized the AAV8 vector encoding a fragment of a monoclonal antibody targeting VEGF showed strong efficacy in patients using the third highest evaluated dose with a 14-letter gain and a mean of 2.8 annualized injections (compared to baseline 9.6 annualized injections) at 2 years in nAMD patients. The efficacy was maintained at year 3 with a 2.4 annualized rate of supplemental anti-VEGF injections and 50% (3/6) of patients did not need an additional anti-VEGF injection [68]. With suprachoroidal delivery, preliminary analysis showed that patients received a mean of 1.2 supplemental injections over 6 months following administration of RGX-314, which represents a 75.9% reduction in the anti-VEGF treatment burden. Moreover, 4 of 14 RGX-314 treated patients received no anti-VEGF injections over 6 months [69]. Phase II clinical trials to evaluate the subretinal delivery of RGX-314 in nAMD (NCT04832724), the suprachoroidal delivery in nAMD (AAVIATE; NCT04514653) and the suprachoroidal delivery in DR (ALTITUDE; NCT04567550) is currently underway.

A second nAMD-based gene therapy, ADVM-022, is comprised of an AAV.7m8 vector that produces aflibercept protein and can be delivered as a single intravitreal injection. In Phase I/II clinical trial (OPTIC; NCT03748784), a single dose of intravitreal ADVM-022 was administered to 30 nAMD patients who were previously treated with anti-VEGF therapy. The results showed that 60% of patients had an injection-free interval beyond 1 year. Patients demonstrated an 85% reduction in annualized injection frequency following a single low dose and a 100% reduction with the high dose. In the OPTIC trial, no evidence of significant intraocular inflammation or hypotony was reported but one patient developed a retinal detachment [70,71]. However, a clinical trial of high-dose AVDM-022 in DME (INFINITY; NCT04418427) was halted due to a case of recalcitrant hypotony.

A Phase I/II gene therapy trial for macular atrophy secondary to AMD (NCT03846193) is also ongoing using GT005 (Gyroscope Therapeutics Limited, London, UK), a recombinant non-replicating AAV encoding human complement factor I (CFI).

#### Non-viral vector gene therapy

Gene therapy is not limited to a viral vector-based approach. In a subset of LCA patients, a deep intronic mutation (c.2991 + 1655A > G) in centrosome protein 290 (CEP290) causes a classic splicing defect and premature truncation codon that clinically presents as early rod degeneration with retention of poorly functional central cones [72,73]. To correct the splicing mutation, a 17-mer antisense RNA-based oligonucleotide named sepofarsen was designed to normalize the mutated messenger RNA and produce a wild-type or normal CEP290 [74]. A Phase I/II clinical trial (NCT03140969) that remains currently under active enrolment reported data from the first 10 patients who received a single intravitreal dose of sepofarsen [74]. Preliminary results at the 3-month follow-up were remarkable for one patient with a large improvement in visual acuity from light perception to 20/400 and four other patients with improvements >15 letters. Patients were noted to have less oculomotor instability and better full-field stimulus testing (FST) thresholds in the treated eyes [75]. Thus far, no severe adverse events have been reported requiring premature termination of the trial. Intraocular inflammation was noted with most adverse events rated to be mild to none.

### Stem cell therapy

Due to the ability to differentiate into various cell lineages with a virtually unlimited number of renewals, stem cells have gained interest as a potential treatment for retinal disease and several studies have been conducted over the past decade. The therapeutic action of stem cells is by replacement or repair of a diseased cell, tissue, or organ [76]. Human embryonic stem cells (hESCs), induced pluripotent stem cells (iPSCs; e.g. from skin fibroblast), and somatic stem cells (e.g. from bone marrow, adipose tissue, and central nervous system cell) have been studied and used for these purposes.

At present, no stem cell therapy for retinal diseases has been approved by FDA. Most human trials are still at an early phase (Phase I or II). The RPE is the major target for current stem cell studies, particularly for GA secondary to AMD and hereditary retinal diseases, such as Stargardt’s macular dystrophy and retinitis pigmentosa (RP). RPE transplantation was first reported in 2012 by using hESC-derived RPE implanted into the subretinal space of nine patients with Stargardt’s macular dystrophy and 9 patients with GA from AMD [77,78]. Among these 18 patients (18 eyes), 8 eyes showed visual improvement of at least 15 letters during the first year after surgery while only one eye had decreased vision of more than 10 letters. One eye developed endophthalmitis [78]. At 4 years, more than half of the patients sustained visual improvements with evidence of possible cellular engraftment. No serious adverse events, such as hyperproliferation, tumorigenicity, or rejection-related inflammation were noted in this study [79]. A report of RPE differentiated from iPSC transplanted into the subretinal space of two nAMD patients showed an intact transplanted sheet, but no visual improvement [80]. Another study using iPSC found mutations in the second patient’s cultured iPSCs and the study was suspended before cell implantation [81]. Some studies are now expanding to central retinal vein occlusion (NCT03981549; using intravitreal autologous bone marrow CD34^+^ stem cell), disciform scar secondary to nAMD (NCT02903576; injecting hESC-RPE into the subretinal space) or other diseases, such as glaucoma and optic atrophy (Stem Cell Ophthalmology Treatment Study; SCOTS; NCT01920867 and NCT03011541). The SCOTS trial also demonstrated improvement in vision following injection of autologous bone marrow-derived stem cells *via* combination routes (e.g. retrobulbar, sub-tenon, intravitreal, subretinal, or intra-optic nerve injections), followed by intravenous injection, in 83% of optic nerve atrophy patients, 63% of AMD patients and 62% of Stargardt’s macular dystrophy patients [82–84]. In the AMD cohort, 32 eyes were treated per protocols (11 eyes in arm 1 [retrobulbar, sub-tenon, and intravenous injection], 19 eyes in arm 2 [retrobulbar, sub-tenon, intravitreal, and intravenous injection], and 2 eyes in arm 3 [subretinal and intravenous injection]). The average visual acuity improvement was 1.68 lines for arm 1, 1.29 lines for arm 2, and 3.5 lines for arm 3. Overall, 20 of 32 eyes (63%) experienced an improvement in LogMAR visual acuity, averaging 27.6% (range, 2.5% to 44.6%; mean, 0.96 LogMAR with standard deviation [SD] of 0.42).

The use of autologous adipose tissue-derived stem cells in the treatment of non-neovascular AMD was also reported in three cases. However, severe vision loss after intravitreal injection of adipose tissue-derived stem cells was observed. The visual acuity ranged from 20/200 to no light perception one year after injection and was associated with ocular hypertension, haemorrhagic retinopathy, vitreous haemorrhage, combined tractional and rhegmatogenous retinal detachment, and/or lens dislocation [85].

### Artificial vision

#### Optogenetics gene therapy

Optogenetics is a biological technique where an ectopically expressed light-sensitive ion channel is genetically engineered into a living cell. As the retina is a light-sensitive organ, the use of optogenetics in the treatment of retinal diseases, particularly those of photoreceptors, is a practical approach. The general strategy is to create light-sensitive cells downstream of photoreceptors in either the middle or inner retina. By genetically modifying the middle or inner retina to be photosensitive, light can activate the visual cortex bypassing the principal level of pathology.

The possibility of optogenetics was initially demonstrated by a seminal paper in 2005 [86]. The infected neurons with channelrhodopsin-2, a light-sensitive cation channel, through lentiviral gene delivery. This allowed for temporally precise, non-invasive activation of targeted neurons. Just like its human homolog rhodopsin, algae-based channelrhodopsin is a photosensitive G-coupled protein receptor that is activated by a specific wavelength of light. In contrast to normal human rhodopsin proteins, channelrhodopsin directly forms an ion channel and allows for depolarization of a neuron [87]. In the retina, this translates into a bipolar cell-activating in response to a photon of light and then signalling to its coupled ganglion cell, creating the potential for meaningful vision. Multi-characteristic opsin (MCO; vMCO-010; Nanoscope Therapeutics Inc., Bedford, TX, USA) therapy is based on an intravitreal injection of virus encoding a light-sensitive ion channel that targets bipolar cells. In the Phase I/IIa study, 11 patients with advanced RP with no light perception were injected with vMCO-010 and the therapy appeared to be well-tolerated. Six of seven patients (86%) with high dose viral titre showed visual acuity improvements of 15 letters or more. The study also reported long-lasting improvements in outdoor light sensitivities and daily activities [88].

Combined with goggles engineered to deliver appropriate light stimulation, a single intraocular injection of an optogenetic vector GS030-Drug Product (PIONEER; NCT03326336; GenSight Biologics, Paris, France) is now currently being investigated in an ongoing trial in RP patients. The goggles detect local changes in light intensity and project corresponding light pulses onto the retina in real-time to activate optogenetically transduced retinal ganglion cells. The preliminary results demonstrated that the patients perceived, located, counted, and touched different objects while at baseline the patient could not visually detect any objects before injection with or without the goggles, or after injection without the goggles. During visual perception, multichannel electroencephalographic recordings revealed object-related activity above the visual cortex. No intraocular inflammation or adverse events were reported. The results from these studies have potentially demonstrated a partial functional recovery in eyes with retinal dystrophy after optogenetic therapy [89].

#### Retinal prostheses

In 1755, Le Roy observed that electrical stimulation of the ocular surface could stimulate the perception of light or phosphenes and in 1929 Foerster found that external stimulation of the occipital lobe could also elicit phosphene perception [90,91]. Since these landmark studies, there has been interest in the possibility of visual prosthetics which have now been extensively studied and developed [90,91]. These prostheses typically consist of a video camera, an image converter transforming light from images to an electrical signal, a small electronic device processing the signal and generating an electrical pulse, and an array of microelectrodes stimulating the retina or other tissue [90,92]. The quality of the perceived image depends on the number of electrodes/photodiodes in the implant, the stimulation strategy implemented and the level of greyscale perception of the patients. Advanced image processing algorithms can also assist some tasks, such as edge detection, and thus highlight the border of objects [91]. Utilizing an intact optic nerve and visual cortex, retinal prosthetic devices are implanted in the eye, above or below the retina, and stimulate the sensation of vision in patients with severe visual impairment, such as those with advanced RP or severely advanced AMD. Retinal prosthetic devices can be primarily classified according to the location of the implant: epiretinal, subretinal, or suprachoroidal.

The epiretinal prosthesis is placed on the inner surface of the retinal nerve fibre layer and offers advantages in that the surgeon may be more familiar with the surgical techniques and explantation, if necessary, is less complex [90,93]. The subretinal prosthesis is placed at the level of degenerated photoreceptors or the outer retina which constitutes a more challenging surgical approach. However, the subretinal location of the arrays provides some benefits, such as lower stimulation thresholds and more stable fixation [93,94]. The suprachoroidal prosthesis is placed between the choroid and the sclera, or in a scleral pocket. This approach offers the advantage of less retinal injury given the absence of direct contact. As it is further from the retina, the suprachoroidal prosthesis requires a higher stimulation threshold which also simultaneously carries a higher risk of damage [93,95].

Many retinal prosthetic devices have been studied over the last few decades in humans. Examples of these prostheses include:

- The Argus II (Second Sight Medical Products Inc., Sylmar, CA, USA), Intelligent Retinal Implant System II (IRIS V2; Pixium Vision S.A., Paris, France), and EPI-RET3 (EPI-RET Project, Aachen, Germany), all of which use an epiretinal approach.

To date, the Argus II is the most widely used device with many peer-reviewed publications. Although an acceptable rate of the adverse event has been reported in IRIS V2 or EPI-RET 3, the studies of long-term safety or effectiveness are limited or underpowered [91,96,97].

- The Alpha IMS and AMS (Retina Implant AG, Reutlingen, Germany), and the Photovoltaic Retinal Implant (PRIMA) bionic vision system (Pixium Vision S.A.), all of which use a subretinal approach.

The Alpha IMS (first generation) was reported to have an acceptable safety profile and was noted to restore daily visual function in 45% of the participants while the Alpha AMS (second generation) was reported to have significant challenges during the surgical implantation [98–100]. The French feasibility study of the PRIMA implant reported that the implant was well-tolerated and provided visual acuity improvement up to 0.9 LogMAR in patients with GA secondary to AMD [101].

- Bionic Vision Australia (BVA; Melbourne, Australia) uses a suprachoroidal approach.

The Phase I (NCT01603576) and Phase II (NCT03406416; the second generation of the device) trials of the suprachoroidal retinal prosthesis in end-stage RP were completed and showed a safety profile with improvement in visual function, daily activities (e.g., motion discrimination, spatial discrimination, localizing object, etc.) and quality of life [102–104]. The Japanese group (Osaka University) also used the suprachoroidal approach by inserting the electrode array in the scleral pocket in 3 advanced RP patients. The group reported no major complications and significant improvement in visual tasks (localization task and table task) in one patient [105].

However, to our knowledge, Argus II is the only prosthesis that has received both Conformitè Europëenne (CE) approval in Europe (2011) and FDA approval (2013) in the US for the treatment of RP [90,106]. Initially, Argus I was comprised of an array of 16 electrodes while Argus II is comprised of a dense 60-electrode array with increased spatial resolution and a larger visual angle [107]. Improvement in orientation and mobility, target localization, shape and object recognition, and reading of letters and short unrehearsed words have been demonstrated in patients implanted with Argus II [106]. Twenty-four of 30 patients (80%) remained implanted with a functioning Argus II at 5 years and performed significantly better with the Argus II *vs.* those without implants (on compared with off) for all tests [108]. The second generation of external hardware (glasses and processing unit; Argus2s) was recently approved by the FDA (2021) to use with Argus II implants although Second Sight has discontinued Argus II implants since 2019 and focussed on cortical implants instead [91,109]. For AMD, the Argus II trial (NCT02227498) has also been completed and the final results are pending. A study of PRIMA for AMD is currently ongoing (NCT03392324).

Prosthetic devices targeting other segments of the visual pathway, such as the optic nerve, lateral geniculate nucleus (LGB), and visual cortex have also been developed and may be of particular benefit in cases where the retina is severely damaged. Orion Cortical Visual Prosthesis System (Second Sight; NCT03344848) and Intracortical Visual Prosthesis (NCT04634383) have ongoing studies with the cortical prostheses.

#### Other sensory substitution devices

Other sensory substitutes or devices (e.g. vOICe, Sound of Vision, or BrainPort) could potentially translate visual information into auditory or tactile information. With some training, these devices have been reported to help vision-impaired patients expand their perception, especially with elevated and dynamic objects [110–112].

## Discussion

Based on the explosion of pharmacotherapeutics and devices over the past two decades, it is clear we are in the midst of a significant era of novel retinal therapies. These innovations have shown remarkable successes in the management of various retinal diseases [113,114] and have partially restored vision in even severely visually-impaired or blind patients. Next-generation anti-VEGF therapies, such as brolucizumab are now FDA-approved and we anticipate several additional agents and approaches to be introduced into the clinical arena in the next few years which will offer more efficacious and durable treatments for our patients, thereby reducing the burden associated with therapy. Other novel therapeutics will offer options and hope to patients with diseases that previously had no treatment, such as inherited retinal dystrophies.

These novel therapeutics, however, are not without challenges and concerns. The availability and access to these treatments are still limited in many countries outside North America. The cost of treatment may also present a significant barrier, for example, in the US, Argus II treatment costs ∼$150,000, and voretigene neparvovec-rzyl treatment costs $450,000 per eye [115,116]. These are important barriers that preclude the integration of these highly innovative therapies into mainstream clinical use, even if they are in existence. Strategies to target these real-world barriers will be critical to maximize the benefits to all of our patients in this golden era of novel retinal therapeutics.

## Data Availability

The authors confirm that the data supporting the findings of this study are available within the article and its supplementary materials.
